# (*E*)-3,3′,4,4′,7,7′,8,8′-Octa­methyl-2*H*,2′*H*-1,1′-bi(cyclo­penta­[*fg*]acenaphthyl­enyl­idene)-2,2′,5,5′,6,6′-hexa­one dichloro­methane monosolvate

**DOI:** 10.1107/S1600536812016091

**Published:** 2012-04-21

**Authors:** Gregory T. McCandless, Andrzej Sygula, Peter W. Rabideau, Steven F. Watkins, Frank R. Fronczek

**Affiliations:** a Department of Chemistry, Louisiana State University, Baton Rouge, LA 70803, USA

## Abstract

The title compound, C_36_H_24_O_6_·CH_2_Cl_2_, is a dimer of two essentially planar (r.m.s., deviations of fitted plane of 14 pyracene C atoms = 0.0539 and 0.0543 Å) tetra­cyclic pyracene frameworks (each with four methyl groups and three carbonyl groups on the peripheral carbon atoms) twisted along a central C=C bond with an angle of 50.78 (3)° at 90 K. There are notably long C*sp* ^2^—C*sp* ^2^ bonds associated with the carbonyl groups, the longest being 1.601 (3) Å between two carbonyl C atoms. There are also intermolecular carbonyl⋯carbonyl interactions of both parallel and antiparallel types, with C⋯O distances in the range 3.041 (3) to 3.431 (2) Å. This compound is of inter­est with respect to the synthesis of fullerene fragments, such as corannulene and semibuckminsterfullerene derivatives (or ‘buckybowls’), and is a side product of the previously reported oxidation reaction. Structural details, such as planarity analysis of fused rings, out-of-plane deviation of substituents, inter­molecular inter­actions, and longer than typical bond lengths, will be discussed as well as comparisons to structurally related compounds.

## Related literature

For the synthesis of fullerene fragments, see the following recent reviews: Tsefrikas & Scott (2006 ▶); Wu & Siegel (2006 ▶); Sygula (2011 ▶). For structurally related compounds, see also: Abdourazak *et al.* (1994 ▶); Sygula *et al.* (1997 ▶); Mehta *et al.* (1999 ▶); Kilway *et al.* (2004 ▶). For a description of the Cambridge Crystallographic Database, see: Allen (2002 ▶). For tables of van der Waals radii, see: Bondi (1964 ▶). For inter­molecular carbonyl group inter­actions, see: Allen *et al.* (1998 ▶). 
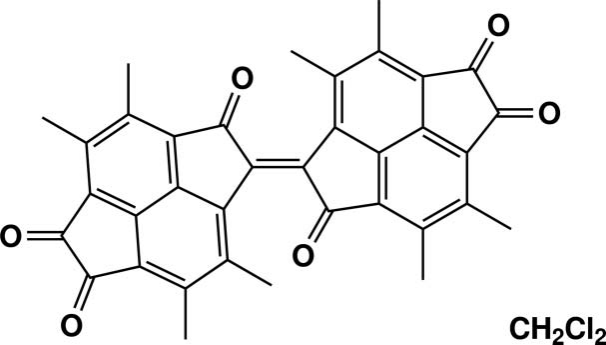


## Experimental

### 

#### Crystal data

C_36_H_24_O_6_·CH_2_Cl_2_
*M* *_r_* = 637.48
Triclinic, 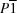

*a* = 8.6644 (15) Å
*b* = 10.959 (2) Å
*c* = 15.856 (3) Å
α = 94.241 (10)°
β = 101.501 (9)°
γ = 95.204 (10)°
*V* = 1462.7 (5) Å^3^
*Z* = 2
Mo *K*α radiation
μ = 0.27 mm^−1^
*T* = 90 K
0.33 × 0.27 × 0.17 mm

#### Data collection

Nonius KappaCCD diffractometer (with an Oxford Cryosystems cryostream cooler)
Absorption correction: multi-scan (*HKL* *SCALEPACK*; Otwinowski & Minor, 1997 ▶) *T*_min_ = 0.915, *T*_max_ = 0.956
12739 measured reflections
6679 independent reflections
5001 reflections with *I* > 2σ(*I*)
*R*_int_ = 0.031

#### Refinement

*R*[*F* ^2^ > 2σ(*F* ^2^)] = 0.045
*wR*(*F* ^2^) = 0.114
*S* = 1.03
6679 reflections
414 parameters
H-atom parameters constrained
Δρ_max_ = 0.8 e Å^−3^
Δρ_min_ = −0.40 e Å^−3^

### 

Data collection: *COLLECT* (Nonius, 2000 ▶); cell refinement: *SCALEPACK* (Otwinowski & Minor, 1997 ▶); data reduction: *DENZO* (Otwinowski & Minor, 1997 ▶) and *SCALEPACK*; program(s) used to solve structure: *SIR97* (Altomare *et al.*, 1999 ▶); program(s) used to refine structure: *SHELXL97* (Sheldrick, 2008 ▶), *SXGRAPH* (Farrugia, 1999 ▶) and *ADDSYM* (Spek, 2003 ▶); molecular graphics: *ORTEP-3 for Windows* (Farrugia, 1997 ▶); software used to prepare material for publication: *SHELXL97* (Sheldrick, 2008 ▶) and *publCIF* (Westrip, 2010 ▶).

## Supplementary Material

Crystal structure: contains datablock(s) global, I. DOI: 10.1107/S1600536812016091/mw2064sup1.cif

Structure factors: contains datablock(s) I. DOI: 10.1107/S1600536812016091/mw2064Isup2.hkl

Additional supplementary materials:  crystallographic information; 3D view; checkCIF report

## Figures and Tables

**Fig. 1.**
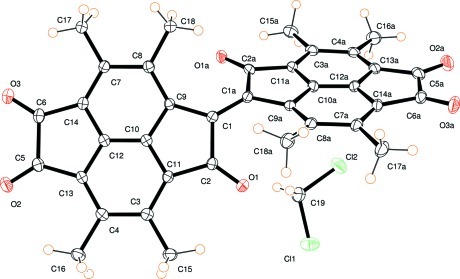
ORTEP representation of (E)-3,3',4,4',7,7',8,8'-octamethyl-2H,2'H-[1,1'-bi(cyclopenta[fg]acenaphthylenylidene)]-2,2',5,5',6,6'-hexaone with 50% probability level atomic displacement ellipsoids at T = 90 K.

**Fig. 2.**
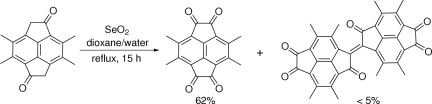
Oxidation of 3,4,7,8-tetramethylcyclopenta[fg]acenaphthylene-1,5(2H,6H)-dione (Sygula et al., 1997).

**Fig. 3.**
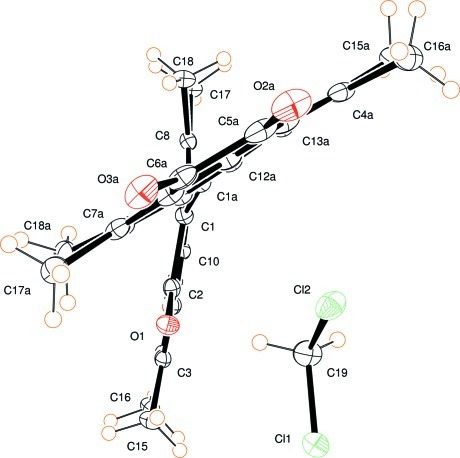
Side profile ORTEP representation showing dihedral angle between the two halves of the title molecule connected by the central C═C bond with 50% probability level atomic displacement ellipsoids at T = 90 K.

**Fig. 4.**
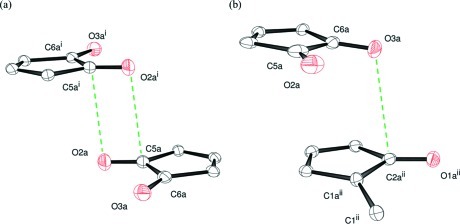
Examples of (a) antiparallel and (b) parallel motifs of intermolecular carbonyl-carbonyl interactions within the crystal structure of this compound. Molecular fragments are shown with connecting dashed lines to help emphasize the difference in two orientations.

## supplementary crystallographic information

### Comment

The structure of title compound (Figure 1 and minor product in Figure 2) can be described as a dimer of two pyracene frameworks joined together with a C═C bond. Both individual tetracyclic subunits are nearly planar (root mean square, or r.m.s., deviation of each fitted plane of 14 pyracene carbon atoms is 0.0539 Å and 0.0543 Å, respectively) and have four methyl groups and three carbonyl groups on the peripheral carbons. The least-squares planes for the two halves of this highly conjugated molecule are twisted along the central C═C bond with an angle of 50.78 (3)° at 90 K. A visual "side-view" *ORTEP* representation of this twist is shown in Figure 3.

The out-of-plane linear deviation of the methyl substituents from a least squares plane of the pyracene carbon atoms ranges from 0.029 (3) Å to 0.365 (3) Å. In comparison to another compound in the Cambridge Structural Database or CSD (Allen, 2002), this range is below the maximum substituent deviation reported for 1,4,5,6,7,10,11,12-octamethylindeno[1,2,3-*cd*]fluoranthene (CSD Refcode NOTVAT) which is a fullerene fragment with 4 methyl groups on the peripheral naphthalene carbons and 4 methyl groups on the peripheral phenyl carbons (Sygula *et al.*, 1997). These methyl carbons were reported to deviate up to 0.4 Å with respect to a fitted least squares plane.

For the planar distortion along the fused bond of one naphthalene subunit, a 6.69 (10)° dihedral angle is calculated between least squares planes of two *ortho*-fused phenyl groups, C3—C4—C13—C12—C10—C11 (r.m.s. deviation of 0.0075 Å) and C7—C8—C9—C10—C12—C14 (r.m.s. deviation of 0.0275 Å). For the other naphthalene subunit, the dihedral angle, 1.53 (12)°, is much smaller between fitted planes of phenyl groups, C7A—C8A—C9A—C10A—C12A—C14A (r.m.s. deviation of 0.0193 Å) and C3A—C4A—C13A—C12A—C10A—C11A (r.m.s. deviation of 0.0226 Å).

The dihedral angle is 3.92 (12)° between two respective least squares planes of the 5-membered carbon rings C5—C6—C14—C12—C13 (r.m.s. deviation of 0.0137 Å) and C1—C2—C11—C10—C9 (r.m.s. deviation of 0.0105 Å) which are connected across the aromatic fused C≐C bond of the naphthalene framework. In a similar comparison to the other half of the compound, there is a significantly larger dihedral angle, 6.57 (14)°, between least squares planes defined by C1A—C2A—C11A—C10A—C9A (r.m.s. deviation of 0.0279 Å) and C5A—C6A—C14A—C12A—C13A (r.m.s. deviation of 0.0147 Å).

An examination of intermolecular carbonyl-carbonyl interactions (Allen *et al.*, 1998) reveals the presence of antiparallel and parallel motifs (Figure 4), but not any perpendicular carbonyl arrangement. The C5a═O2a carbonyls interact with each other in an antiparallel fashion with a torsion angle of zero (C5a═O2a···C5a═O2a) and interatomic distance of 3.041 (3) Å (O2a···C5a). Two different carbonyls, C2a═O1a and C6a═O3a, pack in a parallel relative orientation with -179.65 (17)° torsion angle (C6a═ O3a···C2a═O1a) and interatomic distance of 3.152 (2) Å (O3a···C2a). Close O···C contacts can also be observed among the remaining carbonyls, such as 3.085 (2) Å between O3···C5 and 3.431 (2) Å between O2···C2. However, the torsion angles for these interactions deviate significantly from the expected carbonyl-carbonyl torsion angles. C6═ O3···C5═O2 is pseudo-antiparallel with a torsion angle 63.87 (17)° and C5═O2···C2═ O1 is pseudo-parallel with a torsion angle -112.68 (16)°.

For every equivalent of the title compound, there is an equal molar amount of dichloromethane solvent. A close contacts between one of the solvent's Cl atoms and a carbonyl of the title compound has angles of 167.82 (8)° and 173.25 (15)° for the atoms C19—Cl2···O2a and Cl2···O2a═C5a, respectively, and an interatomic distance between Cl2···O2a of 3.1328 (16) Å, which is less than the sum of the van der Waals radii (Bondi, 1964) of 3.27 Å (*r*_O_ 1.52 Å, *r*_Cl_ 1.75 Å). The angles adopted for this halogen-carbonyl interaction are approaching 180°. The next closest interaction with a halogen and a carbonyl O atom is outside the calculated van der Waals sphere, Cl1···O3 of 3.4166 (16) Å, and deviates away from 180° with a C19—Cl1···O3 angle of 71.06 (8)° and Cl1···O3═C6 angle of 153.92 (13)°.

There are long C(*sp*^2^)—C(*sp*^2^) bond lengths between the carbonyl carbons with a C5—C6 bond length of 1.601 (3) Å and a C5A—C6A bond length of 1.590 (3) Å. These long bond lengths involve 5-membered carbon rings that are fused to a naphthalene framework. The angle between the 3 carbon atoms shared by the 5-membered carbon rings and the naphthalene rings form angles (119.07 (16)° and 119.23 (17)° for C14—C12—C13 and C14A—C12A—C13A, respectively) that are closer to the angles observed in hexagons, 120°, instead of pentagons, 108°. The combination of a long bond length and the deviation in bond angles up to ~119° results in a significantly distorted, yet planar, ring.

An example of this type of ring distortion is found in the structure of 1,2,5,6 tetraketopyracene (Abdourazak *et al.*, 1994), CSD Refcode YEHHAU, which contains two C(*sp*^2^)—C(*sp*^2^) bonds between carbonyl carbons that are both separated by 1.579 (9) Å based on diffraction data collected at T = 115 K. This example also has two enlarged bond angles; both measuring 119.2 (3)° between the fused carbons connecting the 5-membered carbon rings to the naphthalene framework. In the publication containing the 1,2,5,6 tetraketopyracene crystal structure, calculation results were also published and are in good agreement with this structural elongation (using either the PM3 or *ab initio* method).

Also, there are long C(*sp*^2^)—C(*sp*^2^) bond lengths of 1.545 (3) Å for the C1—C2 bond and 1.553 (3) Å for the C1A—C2A bond. These bond lengths are shorter than the bonds discussed in the previous paragraphs. This observation coincides with less bond angle distortion for the carbons in the 5-membered rings that are fused with the naphthalene subunit. The bond angles between fused bonds are 116.98 (16)° and 116.85 (16)° for C9—C10—C11 and C9A—C10A—C11A, respectively, and near the average of 120° (hexagon) and 108° (pentagon).

1,1'-bi(acenaphthen-1-ylidene)-2,2'-dione (CSD Refcode GOZNOY) is structurally similar to the central part of the title compound. The bond distance for C(*sp*^2^)—C(*sp*^2^) bond length between the carbonyl carbon and the carbon connecting the two acenaphthylen-1(2*H*)-one halves is 1.526 (3) Å at T = 295 K (Mehta *et al.*, 1999). A derivative of this diketone compound with the addition of 4 *tert*-butyl groups (CSD Refcode ITILEC) has a C(*sp*^2^)—C(*sp*^2^) bond length of 1.532 (2) Å at T = 200 K (Kilway *et al.*, 2004).

### Experimental

(*E*)-3,3',4,4',7,7',8,8'-octamethyl-2*H*,2'*H*-[1,1'-bi(cyclopenta[*fg*]acenaphthylenylidene)]-2,2',5,5',6,6'-hexaone is a side product of the previously reported oxidation reaction (Figure 2) of 3,4,7,8-tetramethylcyclopenta[*fg*]acenaphthylene-1,5(2*H*,6*H*)-dione with SeO_2_ in a dioxane/water solvent mixture under reflux conditions into the desired 3,4,7,8-tetramethylcyclopenta[*fg*]acenaphthylene-1,2,5,6-tetraone major product (Sygula *et al.*, 1997). The yield of this minor product is < 5% and its crystal structure was not previously published. Single crystals were obtained by recrystallizing in dichloromethane.

### Refinement

All non-hydrogen atoms were identified and subsequently refined anisotropically. With the remaining unaccounted electron densities visible in SXGRAPH (Farrugia, 1999) difference Fourier map, hydrogen atomic sites were generated using HFIX commands and refined as idealized "riding" positions. The extinction parameter had a refined value of zero and, therefore, was omitted from the model. Final refinement cycles included the *SHELXL97* (Sheldrick, 2008) recommended weighting scheme. Missing symmetry was checked using ADDSYM feature in *PLATON* (Spek, 2003).

The highest remaining undetermined electron density above 0.45 e Å^-3^, identified as "Q1", at the conclusion of the refinement in the difference Fourier map is 0.80 e Å^-3^. This electron density is located ~1.2 Å from O2A and "Q1"—O2A—C5A forms an angle of ~100°. "Q1" is also ~1.9 Å from C5A and the angles for "Q1"—C5A—C13A and "Q1"—C5A—C6A are ~91° and ~163°, respectively. No chemically reasonable solution was identified for "Q1".

### Crystal data

**Table:**

C_36_H_24_O_6_·CH_2_Cl_2_	*Z* = 2
*M**_r_* = 637.48	*F*(000) = 660
Triclinic, *P*1	*D*_x_ = 1.447 Mg m^−^^3^
Hall symbol: -P 1	Mo *K*α radiation, λ = 0.71073 Å
*a* = 8.6644 (15) Å	Cell parameters from 6338 reflections
*b* = 10.959 (2) Å	θ = 2.6–27.5°
*c* = 15.856 (3) Å	µ = 0.27 mm^−^^1^
α = 94.241 (10)°	*T* = 90 K
β = 101.501 (9)°	Tabular, red
γ = 95.204 (10)°	0.33 × 0.27 × 0.17 mm
*V* = 1462.7 (5) Å^3^	

### Data collection

**Table:**

Nonius KappaCCD diffractometer (with an Oxford Cryosystems cryostream cooler)	6679 independent reflections
Radiation source: fine-focus sealed tube	5001 reflections with *I* > 2σ(*I*)
Graphite monochromator	*R*_int_ = 0.031
Detector resolution: 9 pixels mm^-1^	θ_max_ = 27.5°, θ_min_ = 2.6°
CCD scans	*h* = −11→11
Absorption correction: multi-scan (*HKL**SCALEPACK*; Otwinowski & Minor, 1997)	*k* = −14→14
*T*_min_ = 0.915, *T*_max_ = 0.956	*l* = −20→20
12739 measured reflections	

### Refinement

**Table:**

Refinement on *F*^2^	Primary atom site location: structure-invariant direct methods
Least-squares matrix: full	Secondary atom site location: difference Fourier map
*R*[*F*^2^ > 2σ(*F*^2^)] = 0.045	Hydrogen site location: inferred from neighbouring sites
*wR*(*F*^2^) = 0.114	H-atom parameters constrained
*S* = 1.03	*w* = 1/[σ^2^(*F*_o_^2^) + (0.0463*P*)^2^ + 0.9955*P*] where *P* = (*F*_o_^2^ + 2*F*_c_^2^)/3
6679 reflections	(Δ/σ)_max_ = 0.001
414 parameters	Δρ_max_ = 0.8 e Å^−^^3^
0 restraints	Δρ_min_ = −0.40 e Å^−^^3^

### Special details

**Table:**

Geometry. All e.s.d.'s (except the e.s.d. in the dihedral angle between two l.s. planes) are estimated using the full covariance matrix. The cell e.s.d.'s are taken into account individually in the estimation of e.s.d.'s in distances, angles and torsion angles; correlations between e.s.d.'s in cell parameters are only used when they are defined by crystal symmetry. An approximate (isotropic) treatment of cell e.s.d.'s is used for estimating e.s.d.'s involving l.s. planes.
Refinement. Refinement of *F*^2^ against ALL reflections. The weighted *R*-factor *wR* and goodness of fit *S* are based on *F*^2^, conventional *R*-factors *R* are based on *F*, with *F* set to zero for negative *F*^2^. The threshold expression of *F*^2^ > σ(*F*^2^) is used only for calculating *R*-factors(gt) *etc*. and is not relevant to the choice of reflections for refinement. *R*-factors based on *F*^2^ are statistically about twice as large as those based on *F*, and *R*- factors based on ALL data will be even larger.

### Fractional atomic coordinates and isotropic or equivalent isotropic displacement parameters (Å^2^)

**Table:**

	*x*	*y*	*z*	*U*_iso_*/*U*_eq_	
Cl1	0.77328 (7)	−0.03949 (5)	0.77593 (4)	0.03263 (15)	
Cl2	0.79071 (6)	0.22106 (5)	0.83069 (4)	0.03157 (15)	
O1	0.29471 (15)	0.01404 (12)	0.78727 (8)	0.0189 (3)	
O2	−0.40015 (16)	−0.17056 (13)	0.40273 (9)	0.0229 (3)	
O3	−0.47615 (16)	0.08134 (13)	0.40444 (9)	0.0241 (3)	
O1A	0.25716 (16)	0.42583 (12)	0.66908 (8)	0.0198 (3)	
O2A	0.93757 (17)	0.58456 (15)	1.06555 (10)	0.0319 (4)	
O3A	0.79850 (17)	0.39242 (15)	1.15279 (9)	0.0278 (4)	
C1	0.1538 (2)	0.18790 (17)	0.73469 (12)	0.0149 (4)	
C2	0.1864 (2)	0.05194 (17)	0.73837 (12)	0.0148 (4)	
C3	0.0352 (2)	−0.14369 (17)	0.63465 (12)	0.0156 (4)	
C4	−0.0979 (2)	−0.17981 (17)	0.56337 (12)	0.0158 (4)	
C5	−0.3252 (2)	−0.08908 (18)	0.45439 (12)	0.0170 (4)	
C6	−0.3647 (2)	0.05076 (18)	0.45374 (12)	0.0175 (4)	
C7	−0.2161 (2)	0.24347 (17)	0.56136 (12)	0.0153 (4)	
C8	−0.0848 (2)	0.27807 (17)	0.63459 (12)	0.0152 (4)	
C9	0.0192 (2)	0.19179 (17)	0.66238 (11)	0.0137 (4)	
C10	−0.0186 (2)	0.06873 (17)	0.62502 (11)	0.0139 (4)	
C11	0.0713 (2)	−0.01902 (17)	0.66389 (11)	0.0143 (4)	
C12	−0.1463 (2)	0.03443 (17)	0.55865 (11)	0.0141 (4)	
C13	−0.1852 (2)	−0.09025 (17)	0.52606 (12)	0.0148 (4)	
C14	−0.2437 (2)	0.12224 (17)	0.52408 (12)	0.0149 (4)	
C15	0.1294 (2)	−0.23962 (18)	0.67640 (13)	0.0213 (4)	
H15A	0.2211	−0.1993	0.719	0.032*	
H15B	0.1659	−0.29	0.6321	0.032*	
H15C	0.0627	−0.2922	0.7053	0.032*	
C16	−0.1435 (2)	−0.31386 (17)	0.53295 (13)	0.0199 (4)	
H16A	−0.2321	−0.3224	0.4829	0.03*	
H16B	−0.1753	−0.3572	0.5797	0.03*	
H16C	−0.0528	−0.3494	0.5164	0.03*	
C17	−0.3239 (2)	0.33712 (18)	0.52803 (13)	0.0206 (4)	
H17A	−0.3994	0.3008	0.4758	0.031*	
H17B	−0.2608	0.4089	0.5142	0.031*	
H17C	−0.3818	0.3627	0.5723	0.031*	
C18	−0.0708 (2)	0.40459 (18)	0.68069 (13)	0.0203 (4)	
H18A	−0.0019	0.4068	0.738	0.031*	
H18B	−0.1761	0.4248	0.687	0.031*	
H18C	−0.0254	0.4647	0.6472	0.031*	
C1A	0.2599 (2)	0.28086 (17)	0.78111 (12)	0.0151 (4)	
C2A	0.3176 (2)	0.39486 (17)	0.73854 (12)	0.0161 (4)	
C3A	0.5858 (2)	0.54462 (18)	0.79525 (13)	0.0192 (4)	
C4A	0.7172 (2)	0.57293 (18)	0.86888 (14)	0.0208 (4)	
C5A	0.8232 (2)	0.51493 (19)	1.03078 (14)	0.0245 (5)	
C6A	0.7485 (2)	0.40834 (19)	1.07795 (13)	0.0204 (4)	
C7A	0.4972 (2)	0.24708 (18)	1.01322 (12)	0.0186 (4)	
C8A	0.3683 (2)	0.21677 (18)	0.93896 (12)	0.0171 (4)	
C9A	0.3677 (2)	0.27995 (17)	0.86501 (12)	0.0156 (4)	
C10A	0.4848 (2)	0.37987 (18)	0.86894 (12)	0.0166 (4)	
C11A	0.4710 (2)	0.44799 (17)	0.79784 (12)	0.0160 (4)	
C12A	0.6036 (2)	0.41108 (18)	0.94072 (12)	0.0180 (4)	
C13A	0.7207 (2)	0.50925 (18)	0.94145 (13)	0.0207 (4)	
C14A	0.6118 (2)	0.34447 (19)	1.01342 (12)	0.0187 (4)	
C15A	0.5742 (2)	0.61827 (19)	0.71843 (14)	0.0238 (5)	
H15D	0.4867	0.5802	0.6723	0.036*	
H15E	0.6736	0.6201	0.6977	0.036*	
H15F	0.5547	0.7025	0.7352	0.036*	
C16A	0.8495 (2)	0.6702 (2)	0.86588 (16)	0.0280 (5)	
H16D	0.9166	0.6892	0.9238	0.042*	
H16E	0.805	0.7447	0.8462	0.042*	
H16F	0.9132	0.64	0.8258	0.042*	
C17A	0.5073 (2)	0.1721 (2)	1.08941 (13)	0.0246 (5)	
H17D	0.6081	0.1969	1.1302	0.037*	
H17E	0.5011	0.0846	1.0695	0.037*	
H17F	0.4194	0.1859	1.1181	0.037*	
C18A	0.2343 (2)	0.12270 (19)	0.94566 (13)	0.0214 (4)	
H18D	0.1407	0.1326	0.9015	0.032*	
H18E	0.2093	0.1345	1.0031	0.032*	
H18F	0.2658	0.0398	0.9367	0.032*	
C19	0.6709 (2)	0.0932 (2)	0.76933 (15)	0.0276 (5)	
H19A	0.6416	0.1108	0.7082	0.033*	
H19B	0.5723	0.0781	0.7913	0.033*	

### Atomic displacement parameters (Å^2^)

**Table:**

	*U*^11^	*U*^22^	*U*^33^	*U*^12^	*U*^13^	*U*^23^
Cl1	0.0365 (3)	0.0235 (3)	0.0423 (3)	0.0051 (2)	0.0160 (3)	0.0086 (2)
Cl2	0.0280 (3)	0.0299 (3)	0.0350 (3)	0.0006 (2)	0.0079 (2)	−0.0095 (2)
O1	0.0185 (7)	0.0184 (7)	0.0186 (7)	0.0039 (5)	−0.0002 (5)	0.0033 (6)
O2	0.0218 (7)	0.0223 (8)	0.0211 (7)	−0.0023 (6)	0.0003 (6)	−0.0035 (6)
O3	0.0206 (7)	0.0259 (8)	0.0221 (8)	0.0027 (6)	−0.0039 (6)	0.0006 (6)
O1A	0.0241 (7)	0.0181 (7)	0.0148 (7)	0.0007 (6)	−0.0010 (5)	0.0013 (5)
O2A	0.0210 (8)	0.0343 (9)	0.0343 (9)	0.0041 (7)	−0.0043 (7)	−0.0110 (7)
O3A	0.0239 (8)	0.0415 (9)	0.0158 (7)	0.0114 (7)	−0.0029 (6)	−0.0009 (6)
C1	0.0144 (9)	0.0153 (9)	0.0149 (9)	0.0016 (7)	0.0027 (7)	0.0021 (7)
C2	0.0145 (9)	0.0168 (9)	0.0131 (9)	0.0009 (7)	0.0027 (7)	0.0029 (7)
C3	0.0178 (9)	0.0160 (10)	0.0139 (9)	0.0016 (7)	0.0049 (7)	0.0018 (7)
C4	0.0184 (9)	0.0148 (9)	0.0147 (9)	−0.0004 (7)	0.0055 (7)	0.0013 (7)
C5	0.0168 (9)	0.0191 (10)	0.0148 (9)	−0.0005 (8)	0.0040 (7)	0.0002 (8)
C6	0.0147 (9)	0.0231 (10)	0.0138 (9)	0.0001 (8)	0.0020 (7)	0.0019 (8)
C7	0.0142 (9)	0.0163 (10)	0.0152 (9)	0.0013 (7)	0.0019 (7)	0.0029 (7)
C8	0.0159 (9)	0.0151 (9)	0.0143 (9)	−0.0004 (7)	0.0029 (7)	0.0018 (7)
C9	0.0147 (9)	0.0141 (9)	0.0119 (9)	0.0000 (7)	0.0023 (7)	0.0016 (7)
C10	0.0145 (9)	0.0145 (9)	0.0129 (9)	0.0000 (7)	0.0039 (7)	0.0014 (7)
C11	0.0151 (9)	0.0164 (9)	0.0117 (9)	0.0020 (7)	0.0030 (7)	0.0022 (7)
C12	0.0151 (9)	0.0154 (9)	0.0120 (9)	−0.0002 (7)	0.0042 (7)	0.0013 (7)
C13	0.0155 (9)	0.0150 (9)	0.0130 (9)	−0.0017 (7)	0.0031 (7)	−0.0006 (7)
C14	0.0131 (8)	0.0176 (10)	0.0133 (9)	0.0004 (7)	0.0018 (7)	0.0022 (7)
C15	0.0289 (11)	0.0156 (10)	0.0185 (10)	0.0045 (8)	0.0016 (8)	0.0017 (8)
C16	0.0257 (10)	0.0150 (10)	0.0185 (10)	0.0003 (8)	0.0039 (8)	0.0020 (8)
C17	0.0184 (10)	0.0178 (10)	0.0226 (10)	0.0029 (8)	−0.0021 (8)	0.0000 (8)
C18	0.0205 (10)	0.0155 (10)	0.0227 (10)	0.0039 (8)	−0.0012 (8)	−0.0003 (8)
C1A	0.0146 (9)	0.0168 (10)	0.0136 (9)	0.0037 (7)	0.0014 (7)	0.0010 (7)
C2A	0.0174 (9)	0.0155 (9)	0.0146 (9)	0.0027 (7)	0.0026 (7)	−0.0026 (7)
C3A	0.0185 (9)	0.0161 (10)	0.0233 (10)	0.0033 (8)	0.0061 (8)	−0.0017 (8)
C4A	0.0137 (9)	0.0159 (10)	0.0313 (11)	0.0032 (8)	0.0033 (8)	−0.0055 (8)
C5A	0.0161 (10)	0.0238 (11)	0.0308 (12)	0.0053 (8)	0.0013 (8)	−0.0109 (9)
C6A	0.0162 (9)	0.0264 (11)	0.0175 (10)	0.0093 (8)	−0.0002 (8)	−0.0039 (8)
C7A	0.0196 (10)	0.0232 (11)	0.0135 (9)	0.0100 (8)	0.0019 (7)	−0.0002 (8)
C8A	0.0181 (9)	0.0190 (10)	0.0145 (9)	0.0063 (8)	0.0027 (7)	−0.0004 (8)
C9A	0.0150 (9)	0.0160 (9)	0.0154 (9)	0.0052 (7)	0.0019 (7)	−0.0018 (7)
C10A	0.0142 (9)	0.0187 (10)	0.0159 (9)	0.0037 (7)	0.0013 (7)	−0.0028 (8)
C11A	0.0155 (9)	0.0148 (9)	0.0167 (9)	0.0029 (7)	0.0014 (7)	−0.0016 (7)
C12A	0.0157 (9)	0.0205 (10)	0.0166 (10)	0.0075 (8)	−0.0003 (7)	−0.0036 (8)
C13A	0.0141 (9)	0.0203 (10)	0.0253 (11)	0.0063 (8)	0.0000 (8)	−0.0076 (8)
C14A	0.0166 (9)	0.0245 (11)	0.0139 (9)	0.0086 (8)	−0.0006 (7)	−0.0036 (8)
C15A	0.0259 (11)	0.0192 (11)	0.0273 (11)	0.0008 (8)	0.0092 (9)	0.0012 (9)
C16A	0.0190 (10)	0.0215 (11)	0.0414 (13)	−0.0001 (8)	0.0033 (9)	0.0003 (10)
C17A	0.0257 (11)	0.0310 (12)	0.0172 (10)	0.0099 (9)	0.0009 (8)	0.0043 (9)
C18A	0.0210 (10)	0.0251 (11)	0.0185 (10)	0.0034 (8)	0.0040 (8)	0.0038 (8)
C19	0.0209 (10)	0.0264 (12)	0.0328 (12)	0.0014 (9)	−0.0001 (9)	0.0017 (9)

### Geometric parameters (Å, º)

**Table:**

Cl1—C19	1.769 (2)	C18—H18A	0.98
Cl2—C19	1.766 (2)	C18—H18B	0.98
O1—C2	1.217 (2)	C18—H18C	0.98
O2—C5	1.210 (2)	C1A—C9A	1.466 (3)
O3—C6	1.205 (2)	C1A—C2A	1.553 (3)
O1A—C2A	1.208 (2)	C2A—C11A	1.507 (3)
O2A—C5A	1.205 (2)	C3A—C11A	1.394 (3)
O3A—C6A	1.211 (2)	C3A—C4A	1.452 (3)
C1—C1A	1.369 (3)	C3A—C15A	1.502 (3)
C1—C9	1.470 (2)	C4A—C13A	1.386 (3)
C1—C2	1.545 (3)	C4A—C16A	1.503 (3)
C2—C11	1.500 (2)	C5A—C13A	1.508 (3)
C3—C11	1.397 (3)	C5A—C6A	1.590 (3)
C3—C4	1.448 (3)	C6A—C14A	1.483 (3)
C3—C15	1.501 (3)	C7A—C14A	1.390 (3)
C4—C13	1.388 (3)	C7A—C8A	1.447 (3)
C4—C16	1.504 (3)	C7A—C17A	1.503 (3)
C5—C13	1.489 (3)	C8A—C9A	1.404 (3)
C5—C6	1.601 (3)	C8A—C18A	1.507 (3)
C6—C14	1.487 (2)	C9A—C10A	1.413 (3)
C7—C14	1.393 (3)	C10A—C12A	1.372 (3)
C7—C8	1.455 (2)	C10A—C11A	1.389 (3)
C7—C17	1.504 (3)	C12A—C14A	1.403 (3)
C8—C9	1.399 (3)	C12A—C13A	1.408 (3)
C8—C18	1.502 (3)	C15A—H15D	0.98
C9—C10	1.415 (3)	C15A—H15E	0.98
C10—C12	1.369 (2)	C15A—H15F	0.98
C10—C11	1.394 (3)	C16A—H16D	0.98
C12—C14	1.407 (3)	C16A—H16E	0.98
C12—C13	1.411 (3)	C16A—H16F	0.98
C15—H15A	0.98	C17A—H17D	0.98
C15—H15B	0.98	C17A—H17E	0.98
C15—H15C	0.98	C17A—H17F	0.98
C16—H16A	0.98	C18A—H18D	0.98
C16—H16B	0.98	C18A—H18E	0.98
C16—H16C	0.98	C18A—H18F	0.98
C17—H17A	0.98	C19—H19A	0.99
C17—H17B	0.98	C19—H19B	0.99
C17—H17C	0.98		
			
C1A—C1—C9	130.36 (17)	O1A—C2A—C11A	128.50 (18)
C1A—C1—C2	121.11 (16)	O1A—C2A—C1A	126.17 (16)
C9—C1—C2	107.09 (15)	C11A—C2A—C1A	105.05 (15)
O1—C2—C11	127.71 (18)	C11A—C3A—C4A	118.41 (18)
O1—C2—C1	125.78 (17)	C11A—C3A—C15A	121.03 (17)
C11—C2—C1	106.15 (15)	C4A—C3A—C15A	120.57 (17)
C11—C3—C4	118.54 (17)	C13A—C4A—C3A	119.75 (18)
C11—C3—C15	121.45 (16)	C13A—C4A—C16A	120.26 (18)
C4—C3—C15	120.00 (17)	C3A—C4A—C16A	119.99 (19)
C13—C4—C3	119.39 (17)	O2A—C5A—C13A	130.6 (2)
C13—C4—C16	120.90 (17)	O2A—C5A—C6A	122.96 (19)
C3—C4—C16	119.67 (17)	C13A—C5A—C6A	106.45 (16)
O2—C5—C13	131.33 (18)	O3A—C6A—C14A	131.1 (2)
O2—C5—C6	122.48 (17)	O3A—C6A—C5A	122.96 (18)
C13—C5—C6	106.18 (15)	C14A—C6A—C5A	105.93 (16)
O3—C6—C14	131.56 (19)	C14A—C7A—C8A	119.88 (17)
O3—C6—C5	122.29 (17)	C14A—C7A—C17A	120.33 (17)
C14—C6—C5	106.12 (15)	C8A—C7A—C17A	119.78 (18)
C14—C7—C8	119.45 (17)	C9A—C8A—C7A	119.36 (18)
C14—C7—C17	120.39 (16)	C9A—C8A—C18A	122.13 (17)
C8—C7—C17	120.13 (17)	C7A—C8A—C18A	118.43 (17)
C9—C8—C7	119.59 (17)	C8A—C9A—C10A	118.45 (17)
C9—C8—C18	122.17 (16)	C8A—C9A—C1A	135.94 (17)
C7—C8—C18	118.17 (17)	C10A—C9A—C1A	105.09 (16)
C8—C9—C10	118.30 (16)	C12A—C10A—C11A	121.38 (18)
C8—C9—C1	135.75 (17)	C12A—C10A—C9A	121.74 (18)
C10—C9—C1	104.98 (16)	C11A—C10A—C9A	116.85 (16)
C12—C10—C11	120.79 (17)	C10A—C11A—C3A	120.23 (17)
C12—C10—C9	121.98 (17)	C10A—C11A—C2A	105.24 (16)
C11—C10—C9	116.98 (16)	C3A—C11A—C2A	134.50 (18)
C10—C11—C3	120.70 (16)	C10A—C12A—C14A	120.61 (18)
C10—C11—C2	104.74 (16)	C10A—C12A—C13A	120.15 (19)
C3—C11—C2	134.36 (17)	C14A—C12A—C13A	119.23 (17)
C10—C12—C14	120.52 (17)	C4A—C13A—C12A	119.78 (17)
C10—C12—C13	120.37 (17)	C4A—C13A—C5A	136.86 (18)
C14—C12—C13	119.07 (16)	C12A—C13A—C5A	103.36 (17)
C4—C13—C12	120.16 (16)	C7A—C14A—C12A	119.69 (17)
C4—C13—C5	135.69 (17)	C7A—C14A—C6A	135.27 (18)
C12—C13—C5	104.14 (16)	C12A—C14A—C6A	104.90 (17)
C7—C14—C12	119.68 (16)	C3A—C15A—H15D	109.5
C7—C14—C6	135.84 (17)	C3A—C15A—H15E	109.5
C12—C14—C6	104.37 (16)	H15D—C15A—H15E	109.5
C3—C15—H15A	109.5	C3A—C15A—H15F	109.5
C3—C15—H15B	109.5	H15D—C15A—H15F	109.5
H15A—C15—H15B	109.5	H15E—C15A—H15F	109.5
C3—C15—H15C	109.5	C4A—C16A—H16D	109.5
H15A—C15—H15C	109.5	C4A—C16A—H16E	109.5
H15B—C15—H15C	109.5	H16D—C16A—H16E	109.5
C4—C16—H16A	109.5	C4A—C16A—H16F	109.5
C4—C16—H16B	109.5	H16D—C16A—H16F	109.5
H16A—C16—H16B	109.5	H16E—C16A—H16F	109.5
C4—C16—H16C	109.5	C7A—C17A—H17D	109.5
H16A—C16—H16C	109.5	C7A—C17A—H17E	109.5
H16B—C16—H16C	109.5	H17D—C17A—H17E	109.5
C7—C17—H17A	109.5	C7A—C17A—H17F	109.5
C7—C17—H17B	109.5	H17D—C17A—H17F	109.5
H17A—C17—H17B	109.5	H17E—C17A—H17F	109.5
C7—C17—H17C	109.5	C8A—C18A—H18D	109.5
H17A—C17—H17C	109.5	C8A—C18A—H18E	109.5
H17B—C17—H17C	109.5	H18D—C18A—H18E	109.5
C8—C18—H18A	109.5	C8A—C18A—H18F	109.5
C8—C18—H18B	109.5	H18D—C18A—H18F	109.5
H18A—C18—H18B	109.5	H18E—C18A—H18F	109.5
C8—C18—H18C	109.5	Cl2—C19—Cl1	110.44 (11)
H18A—C18—H18C	109.5	Cl2—C19—H19A	109.6
H18B—C18—H18C	109.5	Cl1—C19—H19A	109.6
C1—C1A—C9A	128.93 (17)	Cl2—C19—H19B	109.6
C1—C1A—C2A	121.74 (16)	Cl1—C19—H19B	109.6
C9A—C1A—C2A	107.35 (15)	H19A—C19—H19B	108.1
			
C1A—C1—C2—O1	8.3 (3)	C9—C1—C1A—C2A	−34.6 (3)
C9—C1—C2—O1	175.94 (18)	C2—C1—C1A—C2A	129.85 (19)
C1A—C1—C2—C11	−165.24 (17)	C1—C1A—C2A—O1A	15.1 (3)
C9—C1—C2—C11	2.42 (19)	C9A—C1A—C2A—O1A	−179.58 (19)
C11—C3—C4—C13	0.2 (3)	C1—C1A—C2A—C11A	−159.24 (18)
C15—C3—C4—C13	179.21 (18)	C9A—C1A—C2A—C11A	6.1 (2)
C11—C3—C4—C16	−177.50 (17)	C11A—C3A—C4A—C13A	−3.9 (3)
C15—C3—C4—C16	1.5 (3)	C15A—C3A—C4A—C13A	175.87 (19)
O2—C5—C6—O3	3.8 (3)	C11A—C3A—C4A—C16A	175.50 (19)
C13—C5—C6—O3	−177.47 (18)	C15A—C3A—C4A—C16A	−4.7 (3)
O2—C5—C6—C14	−177.87 (17)	O2A—C5A—C6A—O3A	2.4 (3)
C13—C5—C6—C14	0.89 (19)	C13A—C5A—C6A—O3A	−175.95 (19)
C14—C7—C8—C9	4.9 (3)	O2A—C5A—C6A—C14A	−179.79 (19)
C17—C7—C8—C9	−176.94 (17)	C13A—C5A—C6A—C14A	1.9 (2)
C14—C7—C8—C18	−172.07 (17)	C14A—C7A—C8A—C9A	5.1 (3)
C17—C7—C8—C18	6.1 (3)	C17A—C7A—C8A—C9A	−174.21 (18)
C7—C8—C9—C10	−7.9 (3)	C14A—C7A—C8A—C18A	−171.57 (18)
C18—C8—C9—C10	168.99 (17)	C17A—C7A—C8A—C18A	9.1 (3)
C7—C8—C9—C1	−174.67 (19)	C7A—C8A—C9A—C10A	−6.4 (3)
C18—C8—C9—C1	2.2 (3)	C18A—C8A—C9A—C10A	170.20 (18)
C1A—C1—C9—C8	−28.1 (4)	C7A—C8A—C9A—C1A	−176.7 (2)
C2—C1—C9—C8	165.8 (2)	C18A—C8A—C9A—C1A	−0.1 (4)
C1A—C1—C9—C10	163.9 (2)	C1—C1A—C9A—C8A	−28.4 (4)
C2—C1—C9—C10	−2.20 (19)	C2A—C1A—C9A—C8A	167.7 (2)
C8—C9—C10—C12	5.0 (3)	C1—C1A—C9A—C10A	160.4 (2)
C1—C9—C10—C12	175.54 (17)	C2A—C1A—C9A—C10A	−3.5 (2)
C8—C9—C10—C11	−169.25 (17)	C8A—C9A—C10A—C12A	4.2 (3)
C1—C9—C10—C11	1.3 (2)	C1A—C9A—C10A—C12A	177.22 (18)
C12—C10—C11—C3	1.5 (3)	C8A—C9A—C10A—C11A	−173.62 (18)
C9—C10—C11—C3	175.82 (17)	C1A—C9A—C10A—C11A	−0.6 (2)
C12—C10—C11—C2	−174.05 (17)	C12A—C10A—C11A—C3A	5.0 (3)
C9—C10—C11—C2	0.3 (2)	C9A—C10A—C11A—C3A	−177.17 (18)
C4—C3—C11—C10	−0.3 (3)	C12A—C10A—C11A—C2A	−173.30 (18)
C15—C3—C11—C10	−179.24 (18)	C9A—C10A—C11A—C2A	4.5 (2)
C4—C3—C11—C2	173.67 (19)	C4A—C3A—C11A—C10A	−1.1 (3)
C15—C3—C11—C2	−5.3 (3)	C15A—C3A—C11A—C10A	179.16 (18)
O1—C2—C11—C10	−174.99 (19)	C4A—C3A—C11A—C2A	176.7 (2)
C1—C2—C11—C10	−1.65 (19)	C15A—C3A—C11A—C2A	−3.1 (3)
O1—C2—C11—C3	10.4 (3)	O1A—C2A—C11A—C10A	179.6 (2)
C1—C2—C11—C3	−176.3 (2)	C1A—C2A—C11A—C10A	−6.2 (2)
C11—C10—C12—C14	175.13 (17)	O1A—C2A—C11A—C3A	1.7 (4)
C9—C10—C12—C14	1.1 (3)	C1A—C2A—C11A—C3A	175.8 (2)
C11—C10—C12—C13	−2.6 (3)	C11A—C10A—C12A—C14A	177.22 (18)
C9—C10—C12—C13	−176.65 (17)	C9A—C10A—C12A—C14A	−0.5 (3)
C3—C4—C13—C12	−1.3 (3)	C11A—C10A—C12A—C13A	−4.0 (3)
C16—C4—C13—C12	176.37 (17)	C9A—C10A—C12A—C13A	178.34 (18)
C3—C4—C13—C5	−180.0 (2)	C3A—C4A—C13A—C12A	5.0 (3)
C16—C4—C13—C5	−2.3 (3)	C16A—C4A—C13A—C12A	−174.42 (19)
C10—C12—C13—C4	2.5 (3)	C3A—C4A—C13A—C5A	−174.5 (2)
C14—C12—C13—C4	−175.22 (17)	C16A—C4A—C13A—C5A	6.1 (4)
C10—C12—C13—C5	−178.44 (17)	C10A—C12A—C13A—C4A	−1.1 (3)
C14—C12—C13—C5	3.8 (2)	C14A—C12A—C13A—C4A	177.70 (18)
O2—C5—C13—C4	−5.2 (4)	C10A—C12A—C13A—C5A	178.53 (18)
C6—C5—C13—C4	176.2 (2)	C14A—C12A—C13A—C5A	−2.7 (2)
O2—C5—C13—C12	176.1 (2)	O2A—C5A—C13A—C4A	1.6 (4)
C6—C5—C13—C12	−2.54 (19)	C6A—C5A—C13A—C4A	179.8 (2)
C8—C7—C14—C12	1.2 (3)	O2A—C5A—C13A—C12A	−177.9 (2)
C17—C7—C14—C12	−176.97 (17)	C6A—C5A—C13A—C12A	0.2 (2)
C8—C7—C14—C6	176.74 (19)	C8A—C7A—C14A—C12A	−1.4 (3)
C17—C7—C14—C6	−1.4 (3)	C17A—C7A—C14A—C12A	177.92 (18)
C10—C12—C14—C7	−4.2 (3)	C8A—C7A—C14A—C6A	173.6 (2)
C13—C12—C14—C7	173.58 (17)	C17A—C7A—C14A—C6A	−7.1 (3)
C10—C12—C14—C6	179.02 (17)	C10A—C12A—C14A—C7A	−0.9 (3)
C13—C12—C14—C6	−3.2 (2)	C13A—C12A—C14A—C7A	−179.72 (18)
O3—C6—C14—C7	3.3 (4)	C10A—C12A—C14A—C6A	−177.26 (18)
C5—C6—C14—C7	−174.9 (2)	C13A—C12A—C14A—C6A	3.9 (2)
O3—C6—C14—C12	179.3 (2)	O3A—C6A—C14A—C7A	−1.1 (4)
C5—C6—C14—C12	1.16 (19)	C5A—C6A—C14A—C7A	−178.7 (2)
C9—C1—C1A—C9A	163.5 (2)	O3A—C6A—C14A—C12A	174.4 (2)
C2—C1—C1A—C9A	−32.0 (3)	C5A—C6A—C14A—C12A	−3.2 (2)
